# Biology and quality assessment of *Telenomus remus* (Hymenoptera: Scelionidae) and *Trichogramma* spp. (Hymenoptera: Trichogrammatidae) in eggs of *Spodoptera* spp. for augmentative biological control programs

**DOI:** 10.1093/jisesa/iead047

**Published:** 2023-09-18

**Authors:** Alice dos Reis Fortes, Aloisio Coelho, Deoclécio J Amorim, Clarice G B Demetrio, José R P Parra

**Affiliations:** Department of Entomology and Acarology, “Luiz de Queiroz” College of Agriculture (ESALQ), University of São Paulo (USP), Piracicaba, São Paulo, Brazil; Department of Entomology and Acarology, “Luiz de Queiroz” College of Agriculture (ESALQ), University of São Paulo (USP), Piracicaba, São Paulo, Brazil; Department of Exact Sciences, “Luiz de Queiroz” College of Agriculture (ESALQ), University of São Paulo (USP), Piracicaba, São Paulo, Brazil; Department of Exact Sciences, “Luiz de Queiroz” College of Agriculture (ESALQ), University of São Paulo (USP), Piracicaba, São Paulo, Brazil; Department of Entomology and Acarology, “Luiz de Queiroz” College of Agriculture (ESALQ), University of São Paulo (USP), Piracicaba, São Paulo, Brazil

**Keywords:** fall armyworm, isofemale line, egg parasitoid, sustainability

## Abstract

The *Spodoptera* complex of the family Noctuidae, represented here by *S. frugiperda* (J.E. Smith), *S. eridania* (Stoll), *S. albula* (Walker), and *S. cosmioides* (Walker), is an important group of crop pests in Brazil. *Spodoptera frugiperda* and *S. eridania* are invasive in Africa, and the former also in Asia and Oceania. The egg parasitoids *Telenomus remus* Nixon (Hymenoptera: Scelionidae) and *Trichogramma* spp. (Hymenoptera: Trichogrammatidae) are potential control agents for field use against these noctuids. We evaluated the parasitism efficiency, development, and flight capacity of an isofemale line and a regular line of *T. remus*, and 2 genetically variable populations of *Trichogramma pretiosum* Riley and *Trichogramma atopovirilia* Oatman and Platner (Hymenoptera: Trichogrammatidae) in these 4 members of the *Spodoptera* complex. All parasitoids were able to develop in the 4 hosts. The parasitoids showed good flight capacity, except for the regular line of *T. remus.* The *Trichogramma* species, despite having high viability and female:male sex ratios, showed poorer parasitism performances than *T. remus*. The regular *T. remus* line also showed good parasitism capacity and high viability but had a predominance of males. In general, the isofemale line of *T. remus* showed good rates of parasitism and flight capacity as well as a high viability and sex ratio, proving to be a potential candidate for an augmentative biological-control program for *Spodoptera* spp Guenée (Lepidoptera: Noctuidae).

## Introduction

The *Spodoptera* complex of the lepidopteran family Noctuidae, represented here by the species *S. frugiperda* (J.E. Smith), *S. eridania* (Stoll), *S. albula* (Walker), and *S. cosmioides* (Walker), is one of the most important groups of crop pests in Brazil (Parra et al. 2021a). The species have high biotic potentials and cause serious damage to corn (maize), soybean, and cotton crops planted in the no-tillage crop-succession system used in this country (Parra et al. 2021a). In this system, the continuous availability of food allows pests to reproduce year-round, requiring continuous management (Montezano et al. 2018). Recently, *S. eridania* and *S. frugiperda* have been reported in Africa and *S. frugiperda* has also been recorded in India (Goergen et al. 2016, Ganiger et al. 2018, Sharanabasappa et al. 2018) and in Australia (EPPO 2022).

Chemical control and varietal control using transgenic crops with Bt technology are widely used management tactics for *Spodoptera* spp. (Diez-Rodriguez and Omoto 2001, Fatoretto et al. 2017, Barcelos and Angelini 2018). However, irrational use of insecticides tends to select resistant populations (Carvalho et al. 2013) and kill natural enemies, further unbalancing the ecosystem. Therefore, more-sustainable pest-control tactics are essential to reduce the negative effects of agriculture on the environment (Nicholls et al. 1999). In light of current knowledge, one advantage of biological control is the lack of chemical residues that would select resistant pest populations (Norris et al. 2002). Currently, biological pest control is used in many countries (van Lenteren et al. 2017). In Brazil, macro-organisms such as egg parasitoid wasps are used on millions of hectares; for instance, *Trichogramma galloi* Zucchi is used on more than 3 million hectares of sugarcane to control the sugarcane borer *Diatraea saccharalis* (Fabricius) (Parra and Coelho Jr. 2022).

For control of *Spodoptera* spp., especially *S. frugiperda*, the egg parasitoid *Telenomus remus* Nixon is a promising agent. It has been used in small areas in Latin America (Colmenarez et al. 2022). The efficiency of *T. remus* is linked to its ability to parasitize all layers of *Spodoptera* spp. egg masses regardless of their protective scales (Cave 2000). Species of *Trichogramma* can be used to control *Spodoptera*, but are considered less efficient because they parasitize only the surface layers of the egg mass (Beserra and Parra 2004, Cruz and Monteiro 2004).

Most studies with *T. remus* have focused on controlling *S. frugiperda* (Morales et al. 2000, Beserra and Parra 2004, Carneiro et al. 2010, Carneiro and Fernandes 2012, Queiroz et al. 2019) and relatively few have treated other species (Pomari et al. 2012, Pomari-Fernandes et al. 2014). This parasitoid has been recorded naturally in Brazil, i.e., has already adapted to local conditions (Wengrat et al. 2021). On the other hand, *Trichogramma pretiosum* Riley is registered for control of *S. frugiperda* in Brazil (Agrofit 2023). Although the species are relatively well studied, there are discrepancies in the estimated number of individuals to release, and the results for efficiency vary, requiring more-consistent data (Cruz et al. 1999, Cruz and Monteiro 2004, Colmenarez et al. 2022).

Considering the worldwide importance of the *Spodoptera* complex, the often-inefficient conventional control methods make biological control a suitable control alternative. This study evaluated biological and quality control traits of a regular line and an isofemale line, a new approach to attempt to maintain the quality of *T. remus*, as well as regular strains of *T. pretiosum* and *T. atopovirilia* Oatman and Platner as parasitoid agents for the 4 species of the *Spodoptera* complex.

## Material and Methods

### Maintenance Rearing of *Spodoptera* spp.

The rearing colonies of *S. albula*, *S. cosmioides*, *S. eridania*, and *S. frugiperda* were maintained in 2 rooms: the larvae development room and the adult rearing room, with a temperature of 25 ± 2 °C, relative humidity (RH) 60 ± 10%, and 14-h photophase (Parra et al. 2021a). Larvae of *Spodoptera* spp. were reared on an artificial diet developed by Greene et al. (1976).

### Rearing and Maintenance of Parasitoids

A population of *T. remus* with unknown genetic variability, introduced into Brazil from Venezuela in 2010 (Naranjo-Guevara et al. 2020), was studied. This population has been kept for about 6 yr in the Insect Biology Laboratory of the “Luiz de Queiroz” College of Agriculture (ESALQ), University of São Paulo (USP) and in the present study is termed a regular line. The isoline is a *T. remus* population with practically no genetic variability (<14% heterosis and <0.01% DNA variability), that is, an isofemale line established from a population collected in *S. frugiperda* eggs from a cornfield at the ESALQ Areão experimental farm in the municipality of Piracicaba, São Paulo, in 2019 (Wengrat et al. 2021).

In order to establish the isofemale line from the individuals collected in the field, we selected insects with suitable attributes for a biological-control agent, such as good flight capacity and high potential for parasitism. The flight-test unit proposed by Prezotti et al. (2002) was adapted by attaching egg masses of *S. frugiperda* on the upper lid, to be parasitized by flying wasps (de Paula et al. 2022). The flight-selection procedure was carried out for 6 generations. From the insects obtained in the selection of fliers, females were randomly separated and backcrossed for 9 generations, arriving at an insect population with potential heterosis lower than 14% and nuclear DNA variability lower than 0.01% (Li 1955, Coelho et al. 2016).

The parasitoids were kept in 500-ml glass tubes sealed with PVC film. Cards were prepared every 14 days, preferably with 24-h-old egg masses of *S. frugiperda*. These cards were offered to the parasitoids for 72 h and then were removed and transferred to new glass jars containing droplets of pure honey to feed the offspring. Rearing was conducted in a BOD-type climate-control chamber with a temperature of 25 ± 1 °C, RH 60 ± 10%, and 14-h photophase.

The *T. pretiosum* rearing colony was established from parasitized *S. frugiperda* eggs collected in a cornfield on Areão farm at the ESALQ experimental center. This line has been maintained in the laboratory since 2021. *Trichogramma atopovirilia* was collected from eggs of *Helicoverpa zea* (Boddie) in the municipality of São José dos Pinhais, Paraná. The *T. atopovirilia* line has been maintained in a laboratory rearing colony since 2014. To rear these species, we used the protocol described by Parra et al. (2021b). Eggs of the factitious host *Ephestia kuehniella* Zeller were offered for parasitism and development of *Trichogramma* spp., which were sterilized under UV light following the method of Stein and Parra (1987). This rearing colony was kept in a BOD-type climate-control chamber with a temperature of 25 ± 1 °C, RH 60 ± 10%, and 14-h photophase. About 10 days after the eggs were parasitized, the parasitoids emerged and were offered another set of egg masses, restarting the cycle (Parra et al. 2021b).

### Quality Evaluation Experiments of *Telenomus remus* and *Trichogramma* spp.

The performances of the 4 parasitoids, *T. remus* (isofemale line), *T. remus* (regular line), *T. pretiosum*, and *T. atopovirilia* (regular lines), were compared in eggs of *S. frugiperda*, *S. eridania*, *S. albula*, and *S. cosmioides*, in terms of the parasitism rate and development (Supplementary Fig. S1).

For each pest species, 25 recently emerged *T. remus* and *Trichogramma* sp. females (<24 h old) and without experience of parasitism were placed in test tubes (8.5 H × 2.5 D cm). In each test tube, a droplet of pure honey was placed as a food source. Egg masses (<48 h old) of the different species of *Spodoptera*, with about 100 eggs, were separated to assess the parasitism by *Trichogramma* spp., and 250 eggs to evaluate the parasitism by *T. remus*. Egg masses with different numbers of eggs were offered to each parasitoid species according to its parasitism potential. Because *Trichogramma* can parasitize approximately 30–40 eggs in 24 h, we offered fewer eggs to these parasitoids, which made it easier to evaluate and remove the number of larvae hatched. Similarly, a suitable number of eggs was offered to *T. remus*. The egg masses were offered without manipulation, i.e., no layers or scales were removed. The egg masses were attached with Henkel glue on cardboard (5 H × 2 cm) and placed in individual tubes, each containing a female wasp. The tubes were sealed with plastic film.

After 24 h allowed for parasitism, the cards were removed and transferred to other tubes and new cards were offered (Supplementary Table S2). This procedure was continued for 3 consecutive days, a sufficient time span for *T. remus* and *Trichogramma* spp. to parasitize about 75% of the eggs (Schwartz and Gerling 1974, Bueno et al. 2010a, 2010b). The test was carried out under controlled conditions of temperature 25 ± 1 °C, RH 60 ± 10%, and 14-h photophase.

The number of parasitized eggs, viability, sex ratio (females: males), and duration of the egg-to-adult period were evaluated for each parasitoid species and population. For *Trichogramma* spp., the number of individuals emerged per egg was also counted, since these species characteristically superparasitize eggs of *Spodoptera* spp. (Carvalho et al. 2012, Cabezas et al. 2013). To carry out the statistical analyses, the mean of the 3 days of parasitism was used for each biological parameter.

Parasitism was analyzed by counting the number of emerged insects plus the number of parasitized eggs that contained unviable pupae of the parasitoids. The viability of parasitism was determined by calculating the ratio between the number of emerged insects and the number of parasitized eggs. The sex ratio [sr = number of females/ (number of females + number of males)] was determined based on the number of emerged insects.

For the flight tests of *Trichogramma* spp., the flight-test unit developed by Dutton and Bigler (1995) and adapted by Prezotti et al. (2002) was used. A vial containing the insects is placed in the bottom of the flight-test unit, and the glue ring to capture the walking insects is applied 3.5 cm from the bottom of the unit, rather than 2.0 cm (Supplementary Fig. S3). After a 3-day flight period, the test units were transferred to a freezer (–20 °C) to kill the parasitoids and facilitate counting. The parasitoids that did not fly and became immobilized on the glue ring were considered walkers, those that flew and adhered to the Petri dish at the top of the test unit were considered fliers, and those that did not move and remained at the bottom of the test unit were classified as non-fliers. This experiment was carried out under controlled conditions of temperature (25 ± 1 °C), RH 60 ± 10%, and 14-h photophase.

### Data Analysis

Statistical analysis: GLM generalized linear models (Nelder and Wedderburn 1972) with quasi-Poisson distribution were used to analyze parasitism data; GLM with quasi-binomial distribution for sex-ratio data; GLM with binomial distribution to evaluate the viability of parasitism in *S. frugiperda* eggs; GLM with quasi-binomial distribution for the other pest species; and GLM with quasi-binomial distribution for data on *Trichogramma* spp. emerged per egg. The quality of the distribution fits was determined using the half-normal probability graph with simulation envelope of the hnp package (Moral et al. 2017). Means were compared using the Tukey test (*P* = 0.05), especially designed for the GLM of the multcomp package (Hothorn et al. 2008).

Emerged insects were killed in 70% ethanol to count the number of females and males that had emerged that day, and were then discarded. These counts continued until no more wasps emerged. The egg-to-adult period was determined from these data.

The duration of the egg-to-adult period was analyzed using the Shapiro-Wilk analysis of normality, and homogeneity of variances by the Levene test, once the variance assumptions were not accomplished the Kruskal-Wallis test (*P* > 0.05) was adopted. The multiple comparison test, Dunn’s test with Bonferroni correction, was applied to assess differences among treatments (Dunn 1964, Hollander and Wolfe 1973).

Flight-test data were analyzed with a Dirichlet-multinomial model (Kim et al. 2018) to which data for flying, walking, and non-flying individuals were fitted, including the effect of treatment on the linear predictor, using the MGLM package (Zhang and Hua 2018). The likelihood ratio (LR) test was used to assess the significance of the effects between the complete model and the reduced model. The differences among the proportions of individuals in each class, in the different treatments, were evaluated based on bootstrap confidence intervals, with 95% confidence (CI95), assuming normality. To obtain the standard deviations of the predicted proportions, the model was estimated for 10,000 bootstrap samples obtained from the adjusted model, and therefore a parametric bootstrap approach was adopted. Analyses were performed using the R software (R Development Core Team 2021).

To classify the elements (parasitoids × pests) into groups, a table was created with the mean parasitism, viability, sex ratio, egg-to-adult period, and percentage of flying insects of *T. remus* (genetically variable population), *T. remus* (isofemale line), *T. atopovirilia*, and *T. pretiosum* (genetically variable populations) per pest species, *S. albula*, *S. cosmioides*, *S. eridania*, and *S. frugiperda*. Cluster analysis was performed using the Multivariate Analysis package of the R software, using the standardized mean Euclidean distance (average link method, UPGMA). The cophenetic correlation of the group formed was estimated and the significance of this correlation was calculated using the Mantel test.

## Results

The mean number of parasitized eggs differed significantly among the 4 parasitoids, *T. remus* (regular line), *T. remus* (isofemale line), *T. atopovirilia*, and *T. pretiosum* (regular lines), for all *Spodoptera* species evaluated (Supplementary Table S1). For a mean of 3 days of parasitism, in general *T. remus* (regular line) and *T. remus* (isofemale line) parasitized more eggs of the 4 *Spodoptera* species than did the regular lines of the *Trichogramma* species (Table 1). The only exception was *T. remus* (regular line) parasitizing *S. cosmioides* eggs; in this case, the parasitism level was statistically similar to the species of *Trichogramma* (Table 1). For all host species*, T. pretiosum* and *T. atopovirilia* showed similar parasitism potentials (Table 1). The mean numbers of *T. atopovirilia* and *T. pretiosum* emerged per egg of *Spodoptera* spp. did not differ significantly (Supplementary Table S2). Numbers emerged ranged from 1.05 to 1.38 for *T. atopovirilia* and from 1.04 to 1.22 for *T. pretiosum* per egg of the different *Spodoptera* species (Table 2).

**Table 1.** Parasitism of *Telenomus remus* (regular line), *T. remus* (isofemale line), *Trichogramma pretiosum*, and *Trichogramma atopovirilia* in different species of *Spodoptera*, in 72 h. Temperature 25 ± 1 °C, RH 60 ± 10%, and 14-h photophase

*Spodoptera* spp. Parasitoids	*S. albula* ^a^ ^,^ ^b^	*S. cosmioides* ^a^ ^,^ ^b^	*S. eridania* ^a^ ^,^ ^b^	*S. frugiperda* ^a^ ^,^ ^b^
*T. atopovirilia* (regular line)	26.42 ± 4.15 c	19.33 ± 2.72 b	26.56 ± 1.78 b	21.88 ± 2.12 c
*T. pretiosum* (regular line)	28.83 ± 2.63 c	17.26 ± 1.88 b	19.92 ± 2.69 b	21.83 ± 2.36 c
*T. remus* (regular line)	89.26 ± 7.25 a	27.04 ± 3.11 b	65.79 ± 5.39 a	53.00 ± 6.08 b
*T. remus* (isofemale line)	60.47 ± 6.48 b	43.88 ± 5.80 a	49.08 ± 6.31 a	80.75 ± 5.91 a

^a^Mean ± standard error of the mean.

^b^Means followed by the same letter do not differ by Tukey’s test (*P* ≤ 0.05).

**Table 2.** Number of individuals (mean) of *Trichogramma pretiosum* and *Trichogramma atopovirilia* emerged per egg of *Spodopte*ra spp. Temperature 25 ± 1 °C, RH 60 ± 10%, and 14-h photophase

*Spodoptera* spp. parasitoids	*S. albula* ^a^ ^,^ ^b^	*S. cosmioides* ^a^ ^,^ ^b^	*S. eridania* ^a^ ^,^ ^b^	*S. frugiperda* ^a^ ^,^ ^b^
*T. atopovirilia* (variable)	1.10 ± 0.04	1.25 ± 0.06	1.43 ± 0.39	1.16 ± 0.04
*T. pretiosum* (variable)	1.20 ± 0.07	1.31 ± 0.07	1.31 ± 0.09	1.11 ± 0.03

^a^Mean ± standard error of mean.

^b^Nonsignificant difference between column treatments by Tukey’s test (*P* ≤ 0.05).

The parasitism viability differed significantly among the 4 parasitoids with *S. frugiperda*, *S. cosmioides*, and *S. albula* as hosts. The observed parasitism viability of the parasitoids did not differ significantly when the host was *S. eridania* (Supplementary Table S3). In general, parasitism viability was relatively high in all hosts, with levels higher than 90%. However, when *T. remus* (regular line) parasitized *S. albula* eggs, viability was 87.81% (Table 3). In *S. frugiperda*, the parasitism viability of all parasitoid species remained above 95%.

**Table 3.** Parasitism **v**iability (%) of *Telenomus remus* (regular line), *T. remus* (isofemale line), *Trichogramma pretiosum*, and *T. atopovirilia*, in different species of *Spodoptera*. Temperature 25 ± 1 °C, RH 60 ± 10%, and 14-h photophase

*Spodoptera* spp. Parasitoids	*S. albula* ^a^ ^,^ ^b^	*S. cosmioides* ^a^ ^,^ ^b^	*S. eridania* ^a^ ^,^ ^c^	*S. frugiperda* ^a^ ^,^ ^c^
*T. atopovirilia* (regular line)	96.38 ± 1.21 a	93.32 ± 2.12 b	97.53 ± 0.97	95.92 ± 1.56 b
*T. pretiosum* (regular line)	94.43 ± 1.68 a	94.57 ± 1.24 bc	94.14 ± 1.66	98.08 ± 0.62 ab
*T. remus* (regular line)	87.81 ± 2.30 b	99.26 ± 0.29 a	94.33 ± 2.29	95.60 ± 4.16 a
*T. remus* (isofemale line)	97.71 ± 0.60 a	98.28 ± 0.53 ac	92.99 ± 4.12	99.17 ± 0.24 b

^a^Mean ± standard error of the mean.

^b^Means followed by the same letter do not differ by Tukey’s test (*P* ≤ 0.05).

^c^Nonsignificant difference among the means according to Tukey’s test (*P* ≤ 0.05).

The sex ratios of the 4 parasitoids differed significantly in the different host *Spodoptera* species (Supplementary Table S4). The lowest proportions of females were obtained when *T. remus* (regular line) parasitized eggs of *S. cosmioides*, *S. eridania*, and *S. frugiperda*; in this case, the offspring consisted of less than 45% females. The other parasitoids, regardless of the host, maintained percentages of females above 55% (Table 4). The sex ratio of the *T. remus* isofemale line varied depending on the host species, always remaining above 0.69.

**Table 4.** Sex ratios (females: males) of *Telenomus remus* (regular line), *T. remus* (isofemale line), *Trichogramma atopovirilia*, and *Trichogramma pretiosum* when parasitizing eggs of different *Spodoptera* species. Temperature 25 ± 1 °C, RH 60 ± 10%, and 14-h photophase

*Spodoptera* spp. parasitoids	*S. albula* ^a^ ^,^ ^b^	*S. cosmioides* ^a^ ^,^ ^b^	*S. eridania* ^a^ ^,^ ^b^	*S. frugiperda* ^a^ ^,^ ^b^
*T. atopovirilia* (regular line)	0.62 ± 0.05 a	0.70 ± 0.03 a	0.75 ± 0.02 a	0.75 ± 0.03 a
*T. pretiosum* (regular line)	0.56 ± 0.05 b	0.69 ± 0.04 a	0.81 ± 0.03 a	0.68 ± 0.04 a
*T. remus* (regular line)	0.67 ± 0.03 a	0.31 ± 0.06 b	0.40 ± 0.05 b	0.34 ± 0.06 b
*T. remus* (isofemale line)	0.74 ± 0.03 a	0.69 ± 0.04 a	0.79 ± 0.03 a	0.74 ± 0.03 a

^a^Mean ± standard error of the mean.

^b^Means followed by the same letter do not differ by Tukey’s test (*P* ≤ 0.05).

The duration of the egg-to-adult period of the parasitoids differed significantly in the different species of *Spodoptera* (Supplementary Table S5). In all host species, *T. remus* (regular line) and *T. remus* (isofemale line) showed a similar egg-to-adult period of around 14 days (Table 5). For *Trichogramma* spp., the duration of the egg-to-adult period was around 10 days; there was a significant difference only for the host *S. albula*; in this case, the mean egg-to-adult period for *T. atopovirilia* was 10.87 days and for *T. pretiosum* was 9.7 days. The flight capacities of the different parasitoids (*T. remus*, regular line; *T. remus*, isofemale line; *T. pretiosum* and *T. atopovirilia*, regular lines) differed significantly according to the host in which the parasitoids developed (Supplementary Table S6).

**Table 5.** Duration of egg-adult period of *Telenomus remus* (regular line), *T. remus* (isofemale line), *Trichogramma pretiosum*, and *Trichogramma atopovirilia*, when parasitizing different species of *Spodoptera*. Temperature 25 ± 1 °C, RH 60 ± 10%, and 14-h photophase

*Spodoptera* spp. parasitoids	*S. albula* ^a^ ^,^ ^b^	*S. cosmioides* ^a^ ^,^ ^b^	*S. eridania* ^a^ ^,^ ^b^	*S. frugiperda* ^a^ ^,^ ^b^
*T. atopovirilia* (regular line)	10.8 ± 0.12 a	10.23 ± 0.03 b	10.1 ± 0.05 b	9.9 ± 0.04 b
*T. pretiosum* (regular line)	9.7 ± 0.06 b	10.0 ± 0.04 b	10.2 ± 0.08 b	10.2 ± 0.04 b
*T. remus* (regular line)	13.0 ± 0.05 c	14.2 ± 0.06 a	14.3 ± 0.09 a	14.2 ± 0.01 a
*T. remus* (isofemale line)	13.3 ± 0.05 c	13.8 ± 0.04 ba	14.5 ± 0.11 a	14.1 ± 0.04 a

^a^Mean ± standard error of the mean.

^b^Means followed by same letter do not differ by Tukey’s test (*P* ≤ 0.05).

**Fig. 1.**
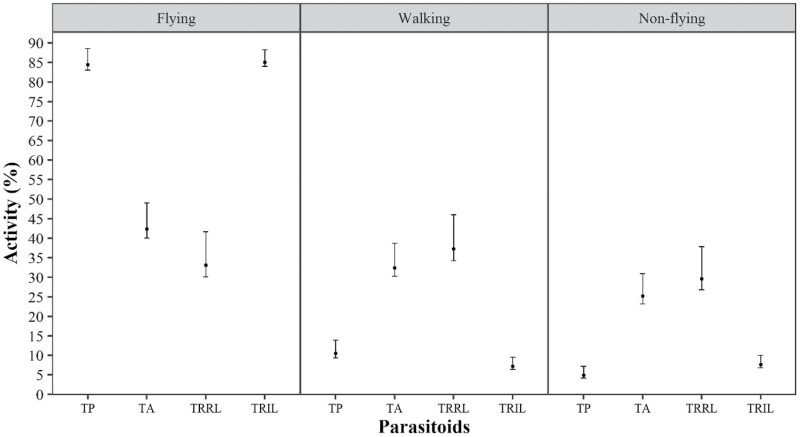
Percentages of flying, walking, and nonflying insects of *Telenomus remus* (regular line – TRRL), *T. remus* (isofemale line – TRIL), *Trichogramma pretiosum* (TP), and *Trichogramma atopovirilia* (TA) (regular line) in eggs of *Spodoptera frugiperda* with respective 95% bootstrap confidence intervals. Temperature 25 ± 1 °C, RH 60 ± 10%, and 14-h photophase.

**Fig. 2.**
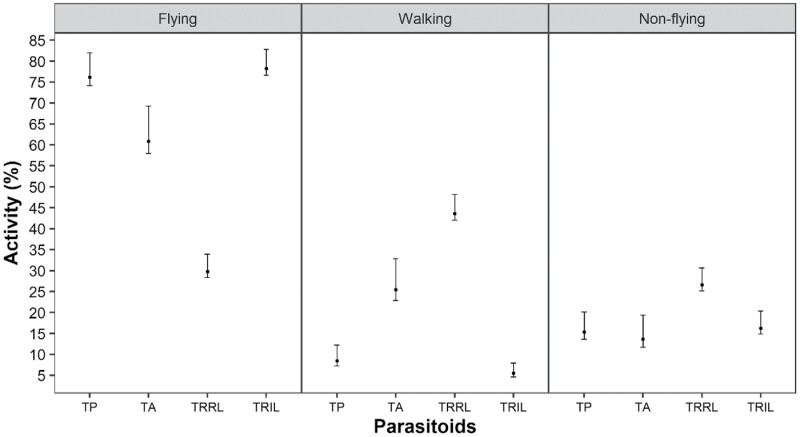
Percentages of flying, walking, and non-flying insects of *Telenomus remus* (regular line – TRRL), *T. remus* (isofemale line – TRIL), *Trichogramma pretiosum* (TP), and *Trichogramma atopovirilia* (TA) (regular line) in eggs of *Spodoptera eridania* in flight test, with respective bootstrap confidence intervals of 95%. Temperature 25 ± 1 °C, RH 60 ± 10%, and 14-h photophase.

**Fig. 3.**
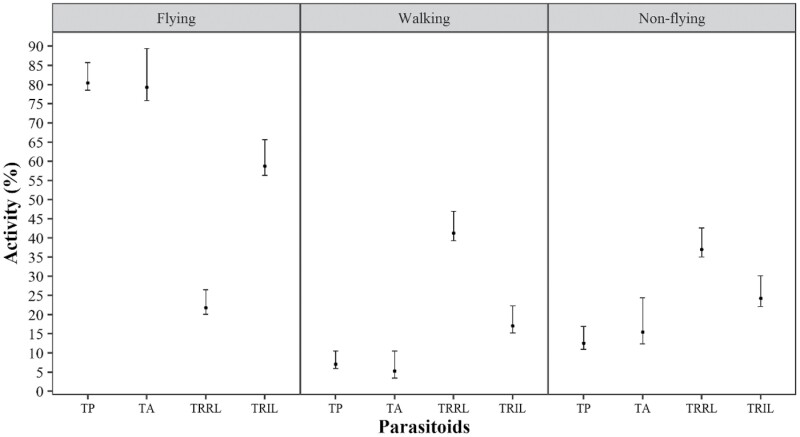
Percentages of flying, walking, and nonflying insects of *Telenomus remus* (regular line – TRRL), *T. remus* (isofemale line – TRIL), *Trichogramma pretiosum* (TP), and *Trichogramma atopovirilia* (TA) (regular line) in eggs of *Spodoptera cosmioides*, in flight test, with the respective bootstrap confidence intervals of 95%. Temperature 25 ± 1 °C, RH 60 ± 10%, and 14-h photophase.

**Fig. 4.**
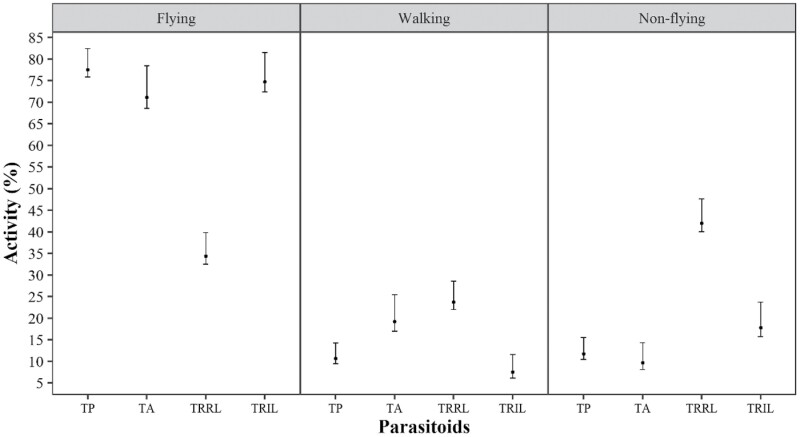
Percentages of flying, walking, and non-flying insects of *Telenomus remus* (regular line – TRRL), *T. remus* (isofemale line – TRIL), *Trichogramma pretiosum* (TP), and *Trichogramma atopovirilia* (TA) (regular line) in *Spodoptera albula* eggs, in flight tests, with the respective bootstrap confidence intervals of 95%. Temperature 25 ± 1 °C, RH 60 ± 10%, and 14-h photophase.

In all species, *T. remus* (isofemale line) and *T. pretiosum* (regular line) maintained a percentage of flying insects higher than 60% (Figs. 1–4). *Trichogramma atopovirilia* showed a percentage of flying insects close to 45% only when the host was *S. frugiperda*; in the other hosts, it maintained a percentage above 60%. The lowest percentages of flying insects were observed for *T. remus* (regular line), always less than 35%, regardless of the host.

In *S. frugiperda* eggs, *T. remus* (isofemale line) and *T. pretiosum* showed the greatest ability to generate flying insects (Fig. 1). Both had essentially the same mean percentage of flying insects, with offspring comprising more than 80% flying individuals (Fig. 1). *Telenomus remus* (regular line) had the lowest percentage of flying insects, 33%, not significantly different from *T. atopovirilia* (regular line), with 42%; consequently, the percentages of walking and non-flying insects increased for these treatments.

In eggs of *S. eridania*, only *T. remus* (regular line) showed a low performance in terms of flight capacity, with approximately 30% of individuals in this category; the percentage of walkers and nonwalkers was higher than 40 and 26%, respectively (Fig. 2). *T. remus* (isofemale line) and *T. pretiosum* (regular line) showed similar percentages of fliers, higher than 75%, differing from *T. atopovirilia*, with about 60% in this category. The proportions of non-flying parasitoids for the treatments *T. pretiosum*, *T. atopovirilia*, and *T. remus* (isofemale line) were statistically equal, around 17%.

In eggs of *S. cosmioides*, the percentage of flying individuals of *T. atopovirilia* and *T. pretiosum* (regular line) was similar, about 80% (Fig. 3). Both differed from *T. remus* (isofemale line), which had about 60% flying individuals. As with the other hosts, *T. remus* (regular line) showed a low potential to generate flying individuals from eggs of *S. cosmioides*, i.e., less than 25% fliers, with 41% walkers and 36% non-fliers. The numbers of non-fliers of *T. pretiosum*, *T. atopovirilia* (regular lines), and *T. remus* (isofemale line) were statistically equal, with a maximum of 25%.

In eggs of *S. albula*, the percentages of flying individuals of *T. remus* (isofemale line), *T. pretiosum*, and *T. atopovirilia* were similar, around 70% (Fig. 4), differing from the percentage of fliers of *T. remus* (regular line), with about 35% fliers. The percentages of non-fliers and walkers were higher for this parasitoid (~42 and 25%, respectively).

**Fig. 5.**
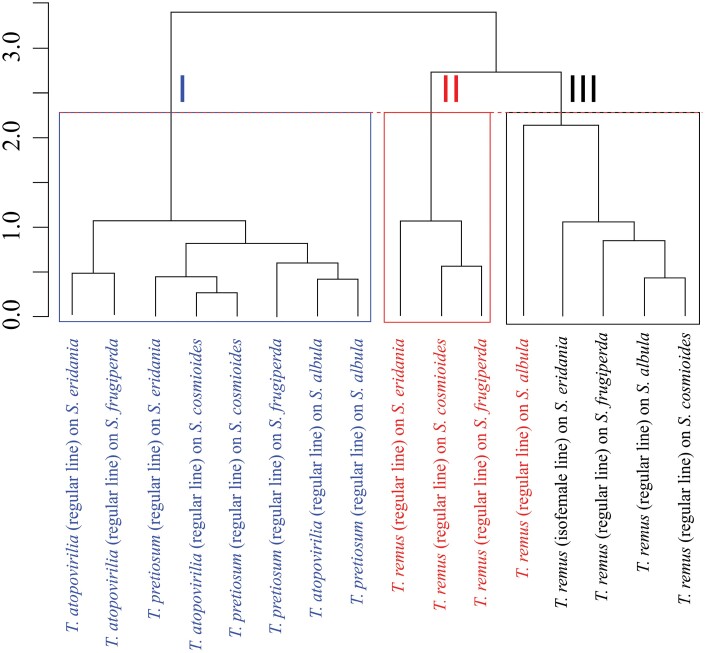
Dendrogram (mean Euclidean Distance-UPGMA) obtained in the cluster analysis for *Telenomus remus* (regular line), *T. remus* (isofemale line), *Trichogramma pretiosum*, and *Trichogrammma atopovirilia* (regular line) in different species of Spodoptera.

The cluster formed has a correlation of 0.76, and according to the Mantel test, this cluster was statistically significant (*P* < 0.001) for the cutoff point by the Mojena method at height 2.25. At the height of 2.25, 3 groups were formed (Fig. 5): (i) one composed of both *Trichogramma* species in all 4 *Spodoptera* species; (ii) one formed by *T. remus* (regular line) in 3 *Spodoptera* species; and (iii) another formed by *T. remus* (isofemale line) plus *T. remus* (regular line) in *S. albula*. Since the parameters of parasitism, viability, sex ratio, and flight capacity were higher and therefore more suitable for a biological-control agent, *T. remus* (isofemale line) proved to be the most promising parasitoid among those evaluated.

## Discussion

Based on the parasitism rate, *T. remus* (both the regular line and the isoline) is more suitable for control of *S. frugiperda*, *S. eridania*, *S. cosmioides*, and *S. albula* than *T. pretiosum* and *T. atopovirilia* (regular lines). The higher capacity for parasitism is due to the physical size of the wasps, which allows *T. remus* to parasitize all layers of the *Spodoptera* egg mass, even though the mass is covered with scales (Cave and Acosta 1999, Cave 2000). The parasitism potential of the smaller-sized *Trichogramma* spp. is impaired by the dense scale covering and the layered arrangement in egg masses of *Spodoptera* spp., so that these parasitoids can parasitize only eggs at the edges and upper layer of the mass, which results in fewer parasitized eggs compared to *T. remus* (Beserra and Parra 2004, 2005, Carneiro and Fernandes 2012). Nevertheless, the *Trichogramma* sp. lines has been reared using *E. kuehniella* eggs. The genetic variation of neither the founder population nor the population used in the experiments was measured. Therefore, we cannot evaluate whether the lines underwent a process of selection on *E. kuehniella-*related haplotypes.

*Telenomus remus*, both the isofemale line and the regular line, showed lower parasitism rates in eggs of *S. cosmioides* than in the other hosts. A possible reason is the volume of eggs of this species (0.033 cm^3^), which is the smallest among the *Spodoptera* species studied here (Supplementary Table S7). However, there are no published reports of this relationship for *T. remus*. It is known only that the size of the egg is related to the amount of food that the parasitoid will have available for development (Godfray 1994). In addition, in *S. cosmioides* the scales deposited on the eggs appear to be larger than the scales produced by the other species, which may also be related to the lower numbers of eggs parasitized by *T. remus* and *Trichogramma* spp., although these hypotheses require evaluation. The parasitism of the *T. remus* regular line was highest on *S. albula* eggs, which caused this line to be grouped with the isoline in the cluster analysis. Pomari et al. (2012) judged *S. albula* to be a suitable host of *T. remus*, although they did not evaluate the parasitism preference.

All 4 host species were suitable for the development of all 4 species of parasitoids, since the viability of parasitism was high, around 90%. According to Pomari et al. (2012), the viability of *T. remus* parasitism for the same host species studied here was also around 90%, although for *S. cosmioides*, the viability dropped to 60%. For *Trichogramma* spp., most studies have focused on the control of *S. frugiperda*; in this host, Beserra and Parra (2004) reported 94% viability for *T. atopovirilia* and *T. pretiosum*, similar to the levels found here, reinforcing the assumption of host adequacy.

The egg-to-adult period did not differ much between the 2 *Telenomus* populations (about 14 days) and between the *Trichogramma* spp. (about 10 days) on the different hosts. According to Vinson (2010), changes in the development time of an insect can indicate better or worse host quality. This again suggests that all the hosts may be nutritionally similar and suitable for the parasitoids.

Another parameter that indicated suitability for control of the *Spodoptera* complex, especially for *T. remus* (isoline), is the sex ratio, which remained above 0.69 (69% females). *Trichogramma* spp. also showed adequate percentages, ranging from 60 to 80%. On the other hand, *T. remus* (regular line) generated less than 40% females in *S. frugiperda*, *S. eridania*, and *S. cosmioides*, indicating a possible selection of haplotypes with lower biotic potential in this wasp population. A higher percentage of females is desirable for use in biological-control programs in the field (Waage and Lane 1984).

Regarding the selection of haplotypes with lower biotic potential of *T. remus* (genetically variable population), another criterion that could strengthen this hypothesis is the ability to fly. This population of *T. remus* showed the lowest percentages, regardless of host species, with less than 35% flying individuals. Haplotype selection, expressed as low flight capacity, can occur when parasitoids are kept in the laboratory for successive generations. For example, in competition within a population, haplotypes that fly less but have greater reproductive vigor will outperform haplotypes that fly more and have less reproductive vigor (Coelho et al. 2016). In this case, the variable population of *T. remus* has been laboratory-maintained for more than 10 yr, explaining the probable selection of haplot9ypes (Naranjo-Guevara et al. 2020). Despite the low flight capacity of *T. remus* (regular line), the other parasitoids, in general, produced a satisfactory percentage of flying insects.

The results for the genetically variable *T. remus* population reinforce the possible advantage of using an isoline, since given its low genetic variability, presumably less than 14% initial heterozygosity and nuclear DNA lower than 0.01%, the chances of selecting haplotypes with less potential as a biological-control agent are lower (Li 1955, Coelho et al. 2016). A molecular-biology study is needed to determine the degree of variability and heterosis potential in the isoline. The isoline studied here was developed using the principles of genetics applied to haplodiploid organisms, no endogamic depression is expected, and the isoline should retain its biological traits over the generations (Sorati et al. 1996, Luna and Hawkins 2004, Prezotti et al. 2004, Coelho Jr. et al. 2016). This technique has been discussed as a method to maintain quality in different natural enemies, including species of the genus *Trichogramma* (Wajnberg and Colazza 1998, Thomson and Hoffmann 2002, Prezotti et al. 2004, Coelho Jr. et al. 2016) and in the family Scelionidae as a way to evaluate patch-time allocation (Wajnberg et al. 2004). As described by Nunney (2003), using an isoline is desirable, although several isolines would be needed in order to maintain the variation (genotypes). However, this is impractical in mass-rearing facilities. Nunney (2003) suggested as an alternative, the selection of specific traits before starting the isoline, as in the case of the present isoline, which shows good flight capacity (de Paula et al. 2022) and parasitism (Lacerda et al. 2022). This pre-selection should ensure that the traits selected for the isoline are suitable for an augmentative biological-control program.

High parasitism capacity and parasitism viability, high sex ratio, and good flight capacity are desirable criteria to select a parasitoid for a biological-control program and are essential parameters to evaluate the quality of insects kept in the laboratory (Parra and Coelho Jr. 2022). Considering all these parameters, the *T. remus* isofemale line proved to be the most suitable for use in a biological-control program for *Spodoptera* spp., with relatively high flight capacity and parasitism on eggs of the *Spodoptera* complex. *Telenomus remus* (regular line) was similar to the *T. remus* isofemale line in most of the traits evaluated; however, the low flight capacity of the regular line renders it unsuitable for use in biological-control programs.

## Supplementary Material

iead047_suppl_Supplementary_Figures

iead047_suppl_Supplementary_Tables
