# Supplementary material for: Biology and quality assessment of Telenomus remus (Hymenoptera: Scelionidae) and Trichogramma spp. (Hymenoptera: Trichogrammatidae) in eggs of Spodoptera spp. for augmentative biological control programs
Source: J Insect Sci. 2023 Sep 18;23(5):5. doi: 10.1093/jisesa/iead047 (PMC10506454; doi:10.1093/jisesa/iead047)
Supplement: iead047_suppl_Supplementary_Figures [file iead047_suppl_supplementary_figures.docx]

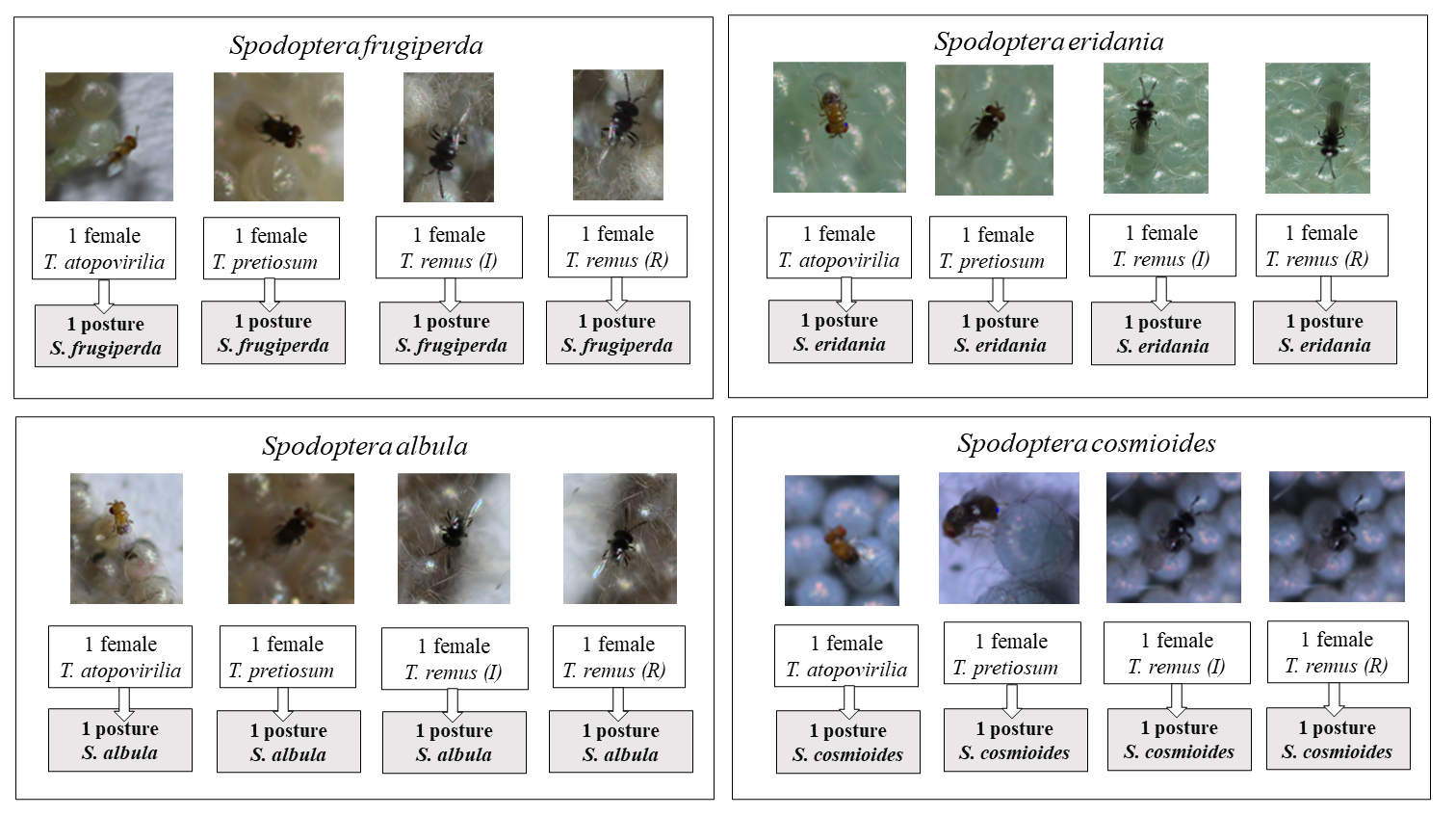


**Fig. S1.** Experimental design for *Telenomus remus* parasitism assay (I- isofemale line), *T. remus* (R- regular line), *Trichogramma pretiosum* (regular line) and *Trichogramma atopovirilia* (regular line) in different species of *Spodoptera*; this scheme refers to one repetition.


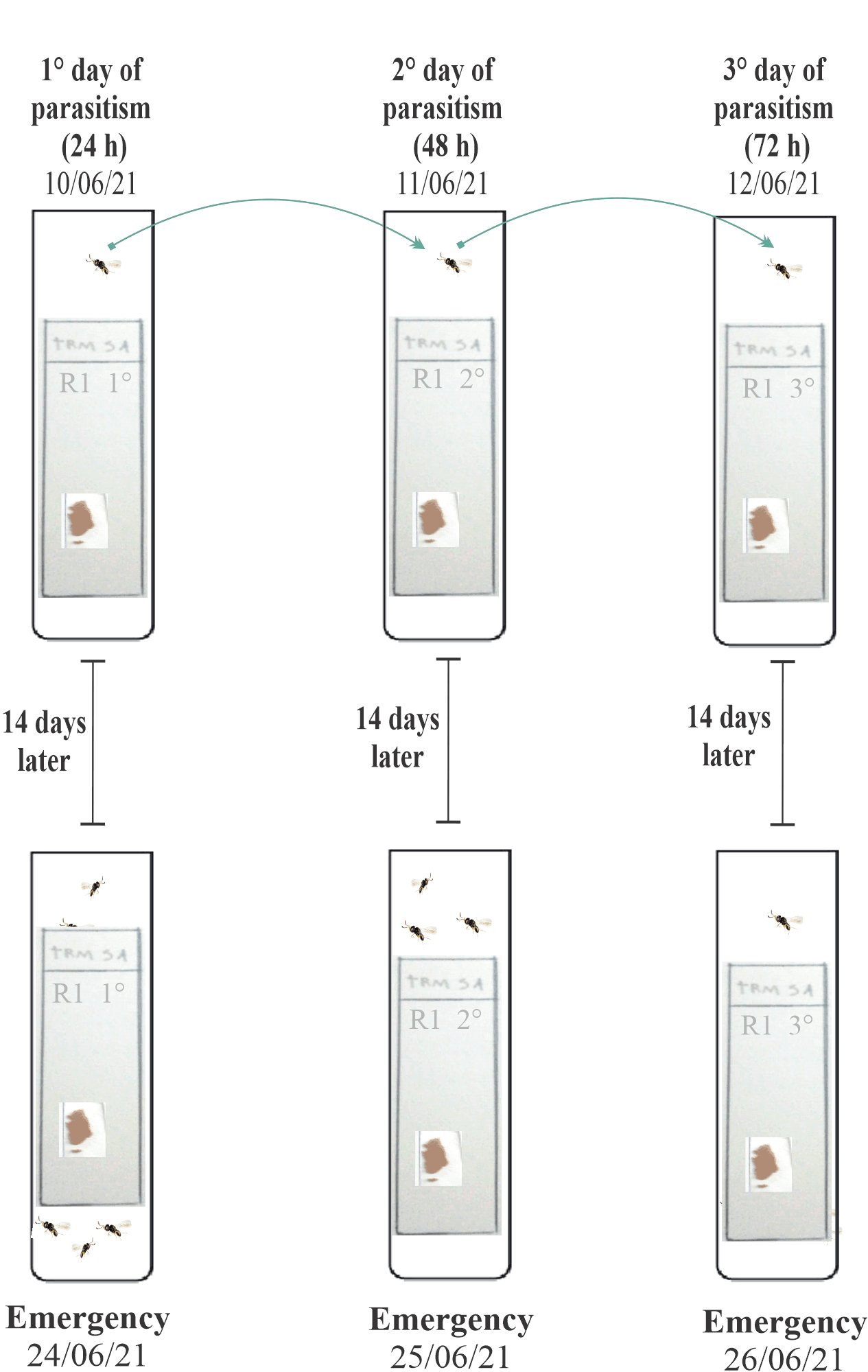


**Fig. S2.** Parasitism process of *Telenomus remus* female on *Spodoptera albula* eggs on the first, second and third days of parasitism; equivalent to one repetition, per day of parasitism.


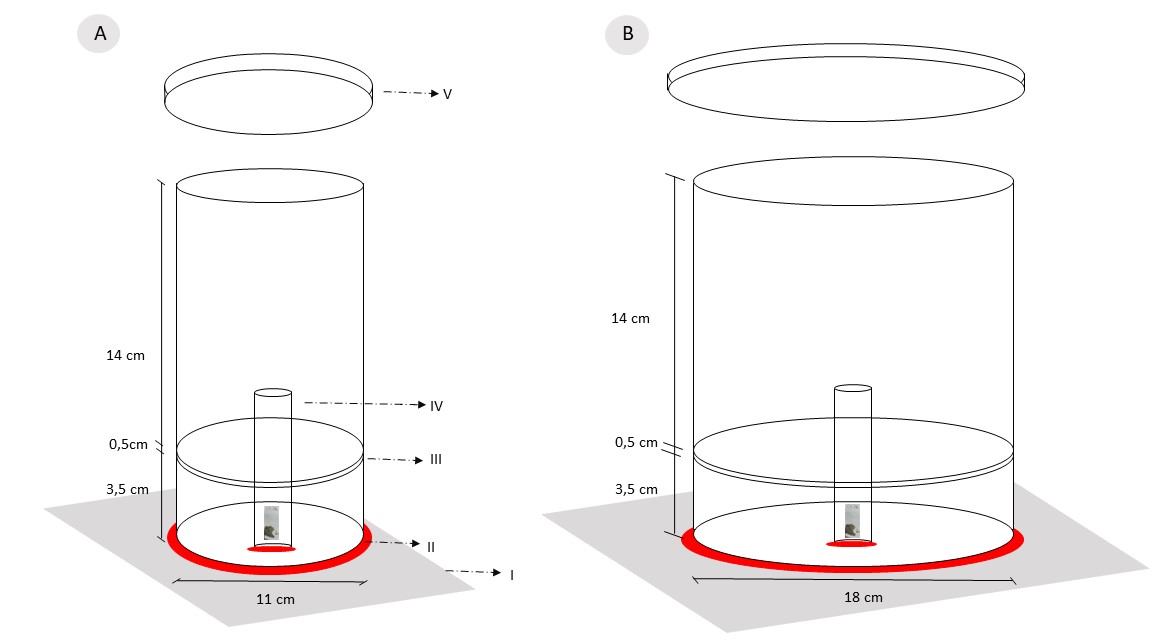


**Fig. S3.** Flight-test units. A. flight unit for *Trichogramma* spp. (I. Medium-density fiberboard, MDF board; II. Non-toxic modeling clay; III. Ring of entomological glue; IV. Test tube containing a parasitized egg mass; V. Petri dish brushed with entomological glue); B. flight unit for *Telenomus remus*.
