# Supplementary material for: Biology and quality assessment of Telenomus remus (Hymenoptera: Scelionidae) and Trichogramma spp. (Hymenoptera: Trichogrammatidae) in eggs of Spodoptera spp. for augmentative biological control programs
Source: J Insect Sci. 2023 Sep 18;23(5):5. doi: 10.1093/jisesa/iead047 (PMC10506454; doi:10.1093/jisesa/iead047)
Supplement: iead047_suppl_Supplementary_Tables [file iead047_suppl_supplementary_tables.docx]

**Table S1.** Statistical parameters obtained through GLM (General Linear Models) for parasitism data of *Telenomus remus* (genetically variable population), *T. remus* (isofemale line), *Trichogramma atopovirilia* (genetically variable population), and *Trichogramma pretiosum* (genetically variable populations) on eggs of *Spodoptera* spp.

| *Spodoptera* spp. | df.^*^ of treatment and residues | F value | p-value |
| --- | --- | --- | --- |
| *S. frugiperda* | 3;92 | 44.988 | <0,001 |
| *S. eridania* | 3;95 | 25.399 | <0,001 |
| *S. cosmioides* | 3;91 | 11.781 | <0,001 |
| *S. albula* | 3;81 | 37.527 | <0,001 |

* df, degrees of freedom

**Table S2**. Statistical parameters of the GLM (General Linear Model) for data on the number of *Trichogramma atopovirilia* (genetically variable population) and *Trichogramma pretiosum* (genetically variable population) emerged from eggs of *Spodoptera* spp.

| *Spodoptera* spp. | | df.^*^ of treatment and residues | | F value | p-value |
| --- | --- | --- | --- | --- | --- |
| *S. frugiperda* | | 1;46 | | 0.2179 | 0.6428 |
| *S. eridania* | | 1;48 | | 2. 1453 | 0,1495 |
| *S. cosmioides* | | | 1;45 | 0.0199 | 0.8884 |
| *S. albula* | 1;41 | | | 0.2645 | 0.6098 |

* df, degrees of freedom

**Table S3.** Statistical parameters of the GLM (General Linear Model) for the parasitism viability data of *Telenomus remus* (genetically variable population), *T. remus* (isofemale line), *Trichogramma atopovirilia* (genetically variable population), and *Trichogramma pretiosum* (genetically variable population) in eggs of *Spodoptera* spp.

| *Spodoptera* spp. | df.^*^ of treatment and residues | F or *X*² value | p-value |
| --- | --- | --- | --- |
| *S. frugiperda* | 3;93 | 31.425 | <0,001 |
| *S. eridania* | 3;95 | 1.009 | 0,3923 |
| *S. cosmioides* | 3;91 | 8,4267 | <0,001 |
| *S. albula* | 3;81 | 163.04 | <0,001 |

* df, degrees of freedom

**Table S4.** Statistical parameters of the GLM (General Linear Model) for sex ratio data of *Telenomus remus* (genetically variable population), *T. remus* (isofemale line), *Trichogramma pretiosum* (genetically variable population), and *Trichogramma atopovirilia* (genetically variable population) emerged from eggs of *Spodoptera* spp.

| *Spodoptera* spp. | DF* of treatment and residues | F value | p-value |
| --- | --- | --- | --- |
| *S. frugiperda* | 3;91 | 20.577 | <0,001 |
| *S. eridania* | 3;94 | 31.286 | <0,001 |
| *S. cosmioides* | 3;91 | 34.451 | <0,001 |
| *S. albula* | 3;81 | 3.3399 | <0,05 |

* df, degrees of freedom

**Table S5.** Statistical parameters of the Kruskal-Wallis analysis when comparing the egg-adult period of *Telenomus remus* (genetically variable population), *T. remus* (isofemale line), *Trichogramma pretiosum* (genetically variable population), and *Trichogramma atopovirilia* (genetically variable population) in eggs of *Spodoptera* spp*.*

| *Spodoptera* spp. | Kruskal-Wallis | p-value |
| --- | --- | --- |
| *S. frugiperda* | 76.74 | <0,001 |
| *S. eridania* | 74,33 | <0,001 |
| *S. cosmioides* | 79,14 | <0,001 |
| *S. albula* | 73,99 | <0,001 |

**Table S6.** Likelihood ratio (LR) test using a Dirichlet-multinomial model, comparing the flight capacity of the four parasitoids reared in different species of *Spodoptera*.

| *Spodoptera* spp. | LR | df^*^ | p-value |
| --- | --- | --- | --- |
| *S. frugiperda* | 100,8201 | 9 | <0,001 |
| *S. eridania* | 88,5994 | 9 | <0,001 |
| *S. cosmioides* | 89,9583 | 9 | <0,001 |
| *S. albula* | 66,4417 | 9 | <0,001 |

*df, degrees of freedom

**Table S7.** Mean length (mm), width (mm), and volume (mm³) of eggs from different species of *Spodoptera* (n = 100).

| *Spodoptera* spp.  Egg measurements | *S. frugiperda* ^1,2^ | *S. eridania*  ^1,2^ | *S. cosmioides* ^1,2^ | *S. albula*  ^1,2^ |
| --- | --- | --- | --- | --- |
| Egg length (mm) | 0,523 ±0,005 | 0,475±0,003 | 0,499±0,004 | 0,483±0,003 |
| Width of eggs (mm) | 0,436±0,004 | 0,368±0,014 | 0,354±0,005 | 0,385±0,005 |
| Volume of eggs (mm³) | 0,052±0,001 a | 0,035±0,002 bc | 0,033±0,001 b | 0,038±0,001 c |

¹ Mean ± standard error of the mean. ² Data analyzed through GLM-Gaussian, means followed by the same letters do not differ by the Tukey test (ANOVA parameters; F_1;48_ = 59,294; p < 0.001).
